# Spread of *Streptococcus pneumoniae* Serotype 8-ST63 Multidrug-Resistant Recombinant Clone, Spain

**DOI:** 10.3201/eid2011.131215

**Published:** 2014-11

**Authors:** Carmen Ardanuy, Adela G. de la Campa, Ernesto García, Asunción Fenoll, Laura Calatayud, Emilia Cercenado, Emilio Pérez-Trallero, Emilio Bouza, Josefina Liñares

**Affiliations:** Hospital Universitari de Bellvitge–Universitat de Barcelona–Fundació Privada Institut d'Investigació Biomèdica de Bellvitge, Barcelona, Spain (C. Ardanuy, L. Calatayud, J. Liñares);; Centros de Investigación Biomédica en Red de Enfermedades Respiratorias, Madrid, Spain (C. Ardanuy, A.G. de la Campa, A. Fenoll, L. Calatayud, E. Cercenado, E. Pérez-Trallero, E. Bouza, E. García, J. Liñares);; Instituto de Salud Carolos II, Madrid (A. G. de la Campa, A. Fenoll);; Consejo Superior de Cientificas, Madrid (A.G. de la Campa, E. García);; Hospital Gregorio Marañón–Universidad Complutense, Madrid (E. Cercenado, E. Bouza);; Hospital Universitario Donostia, Donostia, Spain (E. Pérez-Trallero)

**Keywords:** Streptococcus pneumoniae, bacteria, antimicrobial resistance, multidrug-resistant recombinant clone, fluoroquinolone resistance, Sweden 15A-ST63 clone, serotype 8-ST63, dissemination, spread, Spain

## Abstract

Since 2004, a total of 131 isolates of *Streptococcus pneumoniae* multidrug-resistant invasive serotype 8 have been detected in Spain. These isolates showed resistance to erythromycin, clindamycin, tetracycline, and ciprofloxacin. All isolates were obtained from adult patients and shared a common genotype (sequence type [ST]63; penicillin-binding protein 1a [*pbp1a*]*, pbp2b*, and *pbp2x* gene profiles; *ermB* and *tetM* genes; and a ParC-S79F change). Sixty-eight isolates that required a ciprofloxacin MIC ≥16 μg/mL had additional *gyrA* gene changes. Serotype 8-ST63 *pbp2x* sequences were identical with those of antimicrobial drug–susceptible serotype 8-ST53 isolates. Serotype 8-ST63 *pbp2b* sequences were identical with those of the multidrug-resistant Sweden 15A-ST63 clone. Recombination between the capsular locus and flanking regions of an ST53 isolate (donor) and an ST63 pneumococcus (recipient) generated the novel 15A-ST63 clone. One recombination point was upstream of *pbp2x* and another was within *pbp1a*. A serotype 8-ST63 clone was identified as a cause of invasive disease in Spain.

## Abstract 2

This clone has spread throughout this country and caused invasive pneumococcal disease.

*Streptococcus pneumoniae* is a frequent cause of community-acquired pneumonia, meningitis, bacteremia, and otitis media in children. The diverse biochemical composition of the capsular polysaccharide results in ≥94 serotypes (*1*). However, only a few serotypes cause most invasive disease episodes worldwide. Serotype 8 pneumococci cause invasive pneumococcal disease in adults and have been occasionally associated with outbreaks. Nevertheless, isolates of this serotype are rarely found in children as a cause of invasive disease or as colonizers of the nasopharynx (*2**,**3*). Few lineages have been identified among serotype 8 pneumococci; the major clone is Netherlands 8-ST53, which has been detected worldwide and is typically susceptible to antimicrobial drugs (*4*).

The capsular polysaccharide is the major virulence factor of pneumococci and usually determines their ability to act as invasive or colonizing microorganisms (*3*). In addition to exhibiting the capsule, pneumococci can show low or high genetic diversity, but genotype–serotype association is common. However, this association could be disrupted because of capsular switching caused mainly by recombination of capsular genetic loci. This finding could be a sporadic event, but the recombinant occasionally spreads and could cause pneumococcal disease.

Capsular switching was associated with emergence of a serotype 19A variant of a formerly serotype 4 clone (*5*) related to immunity pressure sustained after a 7-valent pneumococcal conjugate vaccine was introduced into the United States. This well-known phenomenon was the origin of the major penicillin-resistant clone (serotype 14 variant of the Spain 9V-ST156 clone), which caused invasive pneumococcal disease in Spain in the 2000s (*4**,**6*).

Data from the Spanish Reference Laboratory for Pneumococci, which has received pneumococci from Spain since 1979, showed rates of 2.5% to 6.5% for serotype 8 invasive isolates in the last 3 decades. These rates did not show any association with introduction of therapeutic or preventive measures (*7**,**8*). Over these decades, serotype 8 pneumococci were usually susceptible to antimicrobial drugs, although some isolates were resistant to erythromycin or tetracycline. Moreover, serotype 8 pneumococci were isolated mainly from adult patients (*7**,**8*). However, since 2004, serotype 8 pneumococci have been identified that showed resistance to erythromycin, clindamycin, tetracycline, and ciprofloxacin.

In the present study, we analyzed the evolution and molecular epidemiology of these multidrug-resistant serotype 8 pneumococci. We determined whether the increase in these isolates was associated with dissemination of a new recombinant clone (serotype 8-ST63) capable of causing invasive pneumococcal disease in different areas of Spain.

## Materials and Methods

### Bacterial Strains

Isolates received at the Spanish Reference Laboratory for Pneumococci were serotyped by using the quellung reaction and antisera provided by the Staten Serum Institute (Copenhagen, Denmark) (*8*). During January 2004–December 2012, this laboratory received 22,228 invasive pneumococcal isolates (4,274 from children <15 years of age, 16,506 from persons ≥15 years of age, and 448 from persons for whom age data were not available). Of these isolates, 767 were serotype 8 (3.2%). The proportion of serotype 8 was 4.4% among isolates from adults and 0.4% among isolates from children (*8*). Of the 767 serotype 8 pneumococci, 131 isolates were resistant to ≥3 antimicrobial drugs (all isolates were from adults). Of these isolates, 119 were available for molecular characterization.

### Reference Strains

American Type Culture Collection (ATCC) BAA-661, the reference strain of the Sweden 15A-ST63 clone (resistant to erythromycin, clindamycin, and tetracycline, and decreased susceptibility to penicillin), was used as a control for pulsed-field gel electrophoresis (PFGE) and PCR–restriction fragment length polymorphism (PCR-RLFP) analysis of penicillin-binding protein (PBP) genes. Strains CSUB8370, CSUB8757, and CSUB5364 (all 3 strains are serotype 8, susceptible to antimicrobial drugs, and ST53) were also used as controls for PFGE and PCR-RFLP of PBPs (*4**,**9**,**10*).

### Antimicrobial Drug Susceptibility Testing

MICs of antimicrobial drugs for drug-resistant isolates were determined by using the microdilution method, 2%–5% lysed horse blood, and commercially available panels (STRHAE1; Sensititre, East Grunstead, UK) and following the recommendations of the Clinical Laboratory Standards Institute (*11**,**12*). *S. pneumoniae* ATCC 49619 was used for quality control testing. When ciprofloxacin or levofloxacin MICs were >2 μg/mL, these MICs were tested by using the E test (AB Biodisk, Solna, Sweden).

### Molecular Typing

Genomic DNA was embedded in agarose plugs and digested with *Sma*I. Fragments were separated by PFGE in a CHEF-DRIII apparatus (Bio-Rad, Hercules, CA, USA as described (*9*). PFGE patterns were compared visually with patterns of reference and control strains. Seventeen strains selected from different areas and years were studied by using multilocus sequencing typing (MLST) as described (*13*). Allele numbers and sequence types (STs) were assigned by using the pneumococcal MLST website (http://www.mlst.net).

### PBP Fingerprinting

The analysis of *pbp1a*, *pbp2b*, and *pbp2x* genes was performed by using PCR-RFLP. We used primers described by du Plessis et al. (*14*) for *pbp1a* and those described by Gherardi et al. (*15*) for *pbp2b* and *pbp2x*. PCR products were digested with *Hinf*I, and digestion patterns were visually compared. In addition, both strands of PCR PBP gene products of 3 selected strains were purified and sequenced by using amplification primers for *pbp2b* and *pbp2x*. For *pbp1a* amplification, primers and PBP1ASq1 (5′-TAAGGTCTACATGTCTAAT-3′) were used for sequencing purposes.

### Resistance Phenotype Characterization

The presence of macrolide resistance genes (*ermB, mefA*/*E*) and the tetracycline resistance gene (*tetM*) was tested by PCR described elsewhere (*16*). To characterize quinolone resistance for all strains, we used a PCR-RFLP method that detects point mutations at the main quinolone resistance determinant region (QRDR) positions (*17**,**18*). In brief, after PCR amplification, *parC* amplicons were digested with *Hinf*I or *Sfa*NI to detect changes in S79 or D83, respectively. PCR products of *parE* were digested with *Hinf*I to detect changes in D435. Finally, *gyrA* amplicons were digested with *Hinf*I (S81) or *Mbo*II (E85) (*17**,**18*). Control strains with known QRDR mutations were used in each run (*19*).

### PCR Amplification and DNA Sequencing and Analysis

QRDRs sequences were determined for 43 strains (15 with low-level ciprofloxacin resistance [LLCipR] [MIC ≤8 μg/mL] and 28 with high-level ciprofloxacin resistance [HLCipR] [MIC ≥16 μg/mL)]. In brief, *gyrA*, *parC, parE*, and *gyrB* genes were amplified by using PCR and primers and conditions previously described (*19*). PCR products were purified, and both strands were sequenced by using primers for PCR amplification (*19*).

### Analysis of Recombination Site Upstream of *pbp2x*

We used sequence alignments of ≈20 kb located upstream of the *pbp2x* gene of 2 serotype 8 (ST53) strains (2071247 [GenBank accession no. ALBK01000004); and 2081685 [ALBN01000001]), 1 serotype 15A (ST63) strain (GA47179 [AIKX01000002]), and strain G54 (serotype 19F, ST63, CP001015) (*20*) pneumococcal genomes to design 7 sets of PCR primers. These primers were used to identify the putative recombination event upstream of *pbp2x* (Table).

**Table:** Primers used to map the putative recombination event upstream of the *pbp2x* gene of *Streptococcus pneumoniae*, Spain, 2004–2012*

Primer	Sequence (5′→3′)	Position†
F1	aaatccgaagaaggccaaat	278234—278253
R1	Gtacttgagattggcgtgtttg	278834—278813
F2	Tcaatgactgtgatgcctgtt	290699—290719
R2	Tgtcagacaaataggacaaggaga	291315—291292
F3	Gtcaatgacaccaacctcttg	282168—282188
R3	Gctatgagccattctagcaaaga	283037—283015
F4	Tgaatgtaaagacacacgaggaa	273278—273300
R4	Cagtgataacgaataccatacagaa	274128—274104
F5	Cagctctatgaacaccggact	289177—289197
R5	Ttcctagtcgtaaccatcatttca	289927—289904
F6	Ccttggatacgggtattcgtt	287148—287168
R6	Gcagtcgcttgaccttttct	287756—287737
F7	Gtggacaggaagcaaagctc	275192—275211
R7	Ggcagtcagatttgcagaca	276056—276037

*The penicillin-binding protein 2x gene (*pbp2x*) (SPG_0305) of *S. pneumoniae* G54 strain (serotype 19F-ST63; GenBank accession no. CP001015) is located between positions 292638 and 294890 of its genome. F, forward; R, reverse. †Corresponding to the genome of the G54 strain.

## Results

### Description and Spread of Multidrug-Resistant Strain

**Figure 1:**
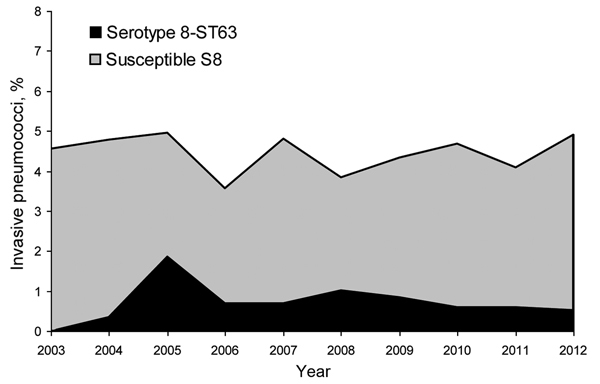
*Streptococcus pneumoniae* serotype 8 among invasive pneumococci isolated from adults in Spain, 2003−2012.

During 2003–2012, among invasive strains collected from adults, the prevalence of serotype 8 ranged between 3.6% and 5.0% (Figure 1). In 2004, four invasive multidrug-resistant serotype 8 isolates were detected at the Spanish Reference Laboratory from 3 hospitals in Madrid (central Spain). Multidrug-resistant serotype 8 isolates were also detected in eastern (2005), southern (2006), and northwestern (2007) Spain. During 2009−2012, multidrug-resistant serotype 8 isolates were detected among invasive isolates from 7 autonomous communities. In the Madrid area, serotype 8-ST63 isolates were detected mainly in adult HIV-infected patients (*21*). All multidrug-resistant serotype 8 isolates were from adults (78.7% from male patients). Sources of isolation of multidrug-resistant serotype 8 pneumococci were blood (n = 116), pleural fluid (n = 6), cerebrospinal fluid (n = 2), fluid from eyes with endophthalmitis (n = 6), and abscess fluid (n = 1).

All 131 multidrug-resistant serotype 8 pneumococci were susceptible to penicillin (MIC <0.03 µg/mL), cefotaxime (MIC <0.03 μg/mL), and amoxicillin (MIC <0.06 μg/mL) and resistant to erythromycin (MIC >128 μg/mL), clindamycin (MIC >128 μg/mL), and tetracycline (MIC >64 μg/mL). Ciprofloxacin MICs ranged from 2 μg/mL to 64 μg/mL, and 68 strains showed HLCipR (MIC ≥16 μg/mL).

### Molecular Typing

**Figure 2:**
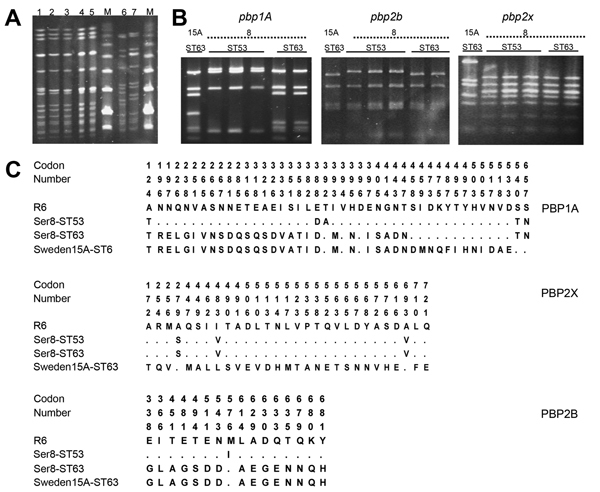
A) Pulsed-field gel electrophoresis patterns of chromosomal DNA of *Streptococcus pneumoniae *isolates after digestion with *Sma*I. Lane 1, Sweden 15A-ST63 (American Type Culture Collection [ATCC] BAA-661); lanes 2−5, serotype 8-ST63; lanes 6 and 7, serotype 8-ST53; lane M, molecular mass markers. B) PCR–restriction fragment length polymorphism patterns of penicillin-binding protein genes *pbp1A, pbp2b*, and *pbp2x*. Lane 1, Sweden 15A-ST63 (ATCC BAA-661) lanes 2−4: serotype 8-ST63; lanes 5 and 6, serotype 8-ST53. C) Amino acid sequence variations among PBP1A, PBP2B, and PBP2X proteins. Row 1, *S. pneumoniae* R6; row 2, serotype 8-ST53; row 3, serotype 8-ST63; row 4, Sweden 15A-ST63 reference strain. Codon numbers of polymorphic sites are numbered in a vertical format. Amino acids are numbered according to their positions in the corresponding protein. Only those amino acids that differ from those of the strain R6 sequence are shown. Dots indicate an amino acid residue that is identical with that in the strain R6 sequence.

A common PFGE pattern (nearly identical to that of the reference Sweden 15A-ST63 clone) was found among all 119 available serotype 8 isolates with multidrug resistance, which suggested a capsular switching event. This PFGE pattern was different from those of other antimicrobial drug–susceptible serotype 8-ST53 isolates (related to the Netherlands 8-ST53 clone) (Figure 2, panel A). These PFGE patterns were confirmed after MLST characterization of 17 selected multidrug-resistant serotype 8 isolates (all were ST63).

### PBP Typing and Analysis

Results of PCR-RFLP analysis of *pbp1A*, *pbp2b*, and *pbp2x* genes of serotype 8-ST63 and antimicrobial drug–susceptible serotype 8-ST53 and of the multidrug-resistant Sweden 15A-ST63 reference strain (ATCC BAA-661) are shown in Figure 2, panel B. Amino acid sequence variation of the PBP1A, PBP2B, and PBP2X proteins of the epidemic strain and 2 control strains is shown in Figure 2, panel C. All serotype 8-ST63 isolates showed the same restriction profile for each *pbp* gene analyzed, which suggested clonal homogeneity. The restriction profile of *pbp2b* of the new strain was identical with that of the Sweden 15A-ST63 clone and different from those of serotype 8-ST53 drug-susceptible strains according to PCR-RFLP results. These results were confirmed after sequencing the *pbp2b* gene amplicons of 3 serotype 8-ST63 isolates, the Sweden 15A-ST63 reference strain, and 2 serotype 8-ST53 isolates (Figure 2, panel C).

The PCR-RFLP profile of *pbp2x* of serotype 8-ST63 isolates was identical with those of serotype 8-ST53 isolates (related to the Netherlands 8-ST53 clone) and different from that of the Sweden 15A-ST63 reference strain. These results were confirmed after sequencing the *pbp2b* gene amplicons of 3 serotype 8-ST63 isolates, the Sweden 15A-ST63 reference strain, and the serotype 8-ST53 isolates.

**Figure 3:**
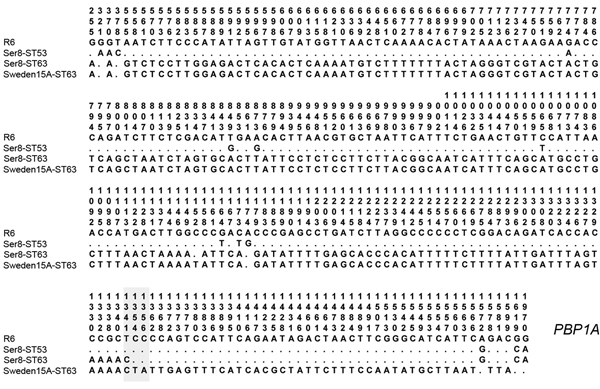
Nucleotide sequence variations in the penicillin-binding protein 1A (*pbp1a*) gene of *Streptococcus pneumoniae*. Row 1, *S. pneumoniae* R6; row 2, serotype (Ser) 8-ST53; row 3, serotype 8-ST63; row 4, Sweden 15A-ST63 reference strain. Polymorphic sites are numbered in a vertical format. Nucleotides are numbered according to their positions in the gene. Only polymorphic sites are shown. Dots indicate a nucleotide that is identical to that in the R6 sequence. The putative recombination site is shaded in gray.

All serotype 8-ST63 isolates had the same PCR-RFLP profile for *pbp1a*. This profile was different from those of antimicrobial drug–susceptible serotype 8-ST53 and nearly identical with the profile of Sweden 15A-ST63. Nucleotide sequence variation in the *pbp1a* gene of serotype 8-ST53, serotype 8-ST63, and Sweden 15A-ST63, determined by using *S. pneumoniae* R6 as a reference, is shown in Figure 3. Using R6 nomenclature for the *pbp1a* gene, we found that sequences of serotype 8-ST63 and Sweden 15A-ST63 were identical for nt 1–1341. Sequences of serotype 8-ST53, serotype 8-ST63, and Sweden 15A-ST63 were identical at nt 1342–1353. Sequences of *pbp1a* for nt 1354–1970 of serotype 8-ST63 were identical with those of antimicrobial drug–susceptible serotype 8-ST53. These results suggest that a recombination point was located in the 3′ end of the *pbp1a* gene.

### Recombination Event Upstream of *pbp2x*

To determine the position of the recombination event that gave rise to the serotype 8-ST63 strain, we searched the public databases for genomic sequences located upstream of the *pbp2x* gene corresponding to serotype 8-ST53 and serotype 15A-ST63 pneumococcal strains. Draft genomic sequences of 2 serotype 8-ST53 strains (2071247 [ALBK01000004]) and 2081685 [ALBN01000001]) were found. These sequences differed only at 5 positions in an ≈20-kb region. The draft sequence of the genome of a single serotype 15A-ST63 (strain GA47179 [ AIKX01000002]) was also used. Moreover, when we compared this sequence (serotype 15A-ST63) with complete genomes of *S. pneumoniae* strains, we found an excellent match with that of strain G54 (serotype 19F; ST63); only 2 nt changes occurred in a 20,792-bp overlap.

**Figure 4:**
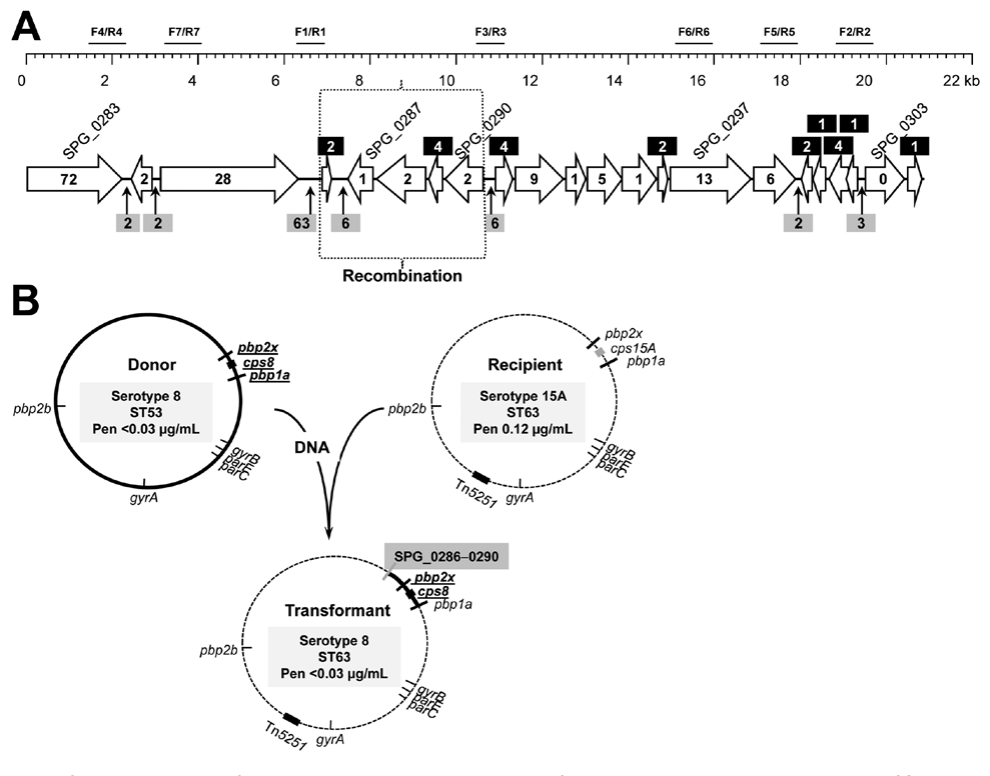
Chromosome location of the recombination region located upstream of the penicillin-binding protein 2x (*pbp2x*) gene of *Streptococcus pneumoniae* (A) and antimicrobial drug resistance determinants, capsular loci of the putative donor, recipient, and recombinant strains (B). In the genomic region upstream of *pbp2x* (SPG_0305), genes have been named as in the G54 genome (ST63) (GenBank accession no. CP001015). Number of polymorphic sites located either within the genes (inserted into the arrows or into black boxes) or in their intergenic regions (gray boxes) is shown. The approximate location of the recombination event is indicated by the dotted box. Amplified and sequenced fragments are indicated in relationship to primer pair designations (Table). MICs for penicillin (Pen) are shown.

The G54 strain has been shown to be a type 19F transformant of a serogroup 15 isolate (*20*). Nucleotide alignment of the 4 strains mentioned above showed several polymorphic sites, which enabled clear (although somehow limited) discrimination between ST53 and ST63 strains (Figure 4, panel A). Different fragments of the ≈20-kb region located upstream of *pbpx2* were amplified by PCR and sequenced (Table; Figure 4, panel A). Sequence comparisons showed that the recombination event between the donor DNA of a serotype 8 (ST53) strain and a recipient strain of serotype 15A (ST63) had occurred in a ≈3.8-kb region that contained genes SPG_0286 and SPG_0290 (using the strain G54 notation). Further attempts to determine more precisely the location of the recombination event were not made because this region has only 17 polymorphic sites. A scheme of the possible recombination event in which capsular loci and flanking regions (*pbp2x* and *pbp1a*) of a serotype 8-ST53 strain were acquired by ST63 pneumococci is shown in Figure 4, panel B.

### Molecular Characterization of Drug Resistance

The *ermB* gene (which confers the macrolide–lincosamide–streptogramin B phenotype) and the *tetM* gene (which confers tetracycline resistance) were detected by PCR. Both genes are usually present in pneumococci related to the Sweden 15A-ST63 clone (*16*). These genes are located in the composite element Tn*5251* (Figure 4, panel B) (*20*).

Among 131 serotype 8-ST63 strains, 63 showed LLCipR (MICs 2 μg/mL–8 μg/mL) and 68 showed (HLCipR (MICs ≥16 μg/mL). An increase in the proportion of serotype 8-ST63 isolates with HLCipR was observed: from 19.5% (8/41) during 2004−2006 to 86.7% (26/30) during 2010−2012 (p<0.001). PCR-RFLP analysis showed that all 119 available serotype 8-ST63 isolates that were studied lacked the restriction site for *Hin*fI in *parC*, which is associated with a change in S79. This change was confirmed to be an S79F after sequencing 43 isolates (15 LLCipR and 28 HLCipR). Fifty-nine of 61 available LLCipR isolates (all with the S79 change) had levofloxacin MICs ≤2 μg/mL (susceptible).

A total of 58 of 68 HLCipR isolates were available for analysis. Of these 58 isolates, 47 lacked the *Hin*fI restriction site in *gyrA*, which is associated with a mutation at S81; 10 isolates lacked the *Mbo*II restriction site related to a *gyrA* change at E85; and 1 isolate lacked the *Hin*fI and *Mbo*II restriction sites that were assumed to be caused by changes in S81 and E85 in *gyrA.* The results of *gyrA* QRDR sequencing of 28 selected HLCipR isolates showed various *gyrA* changes (S81F [n = 23]; S81Y [n = 2]; E85K [n = 3]; and S81F and E85K [n = 1 each]). No *parE* changes were detected by PCR-RFLP or sequencing. Serotype 8-ST63 isolates for which QRDRs were sequenced showed polymorphisms for *parC* (G77[GGA]), *parE* (I460V), and *gyrA* (Y75[TAT]) that were identical with those of the Sweden 15A-ST63 reference strain and other pneumococci of this lineage (*21**,**22*).

## Discussion

In this study, we described the emergence and spread of a pneumococcal clone that originated by recombination of a ciprofloxacin-resistant and multidrug-resistant strain related to the Sweden 15A-ST63 clone, which acted as a recipient of DNA, and a serotype 8 strain with ST53, which acted as a donor of DNA. The new recombinant clone (serotype 8-ST63) was initially confined to the metropolitan area of Madrid and was mainly associated with HIV-infected patients (*21*). However, in the past 5 years, the new clone has been detected in 9 other regions in Spain, which indicates an ability to spread. Two characteristics merged in this new clone, the invasive disease potential of serotype 8 and the antimicrobial drug resistance of ST63, which suggested that the recombinant could be a new antimicrobial drug–resistant clone.

The capsular polysaccharide is the major factor in determining the invasive disease potential of a given isolate. Serotype 8 has been associated with high invasive disease potential when a large collection of invasive and colonizing isolates were compared (*23*). Moreover, capsular serotype 8 was associated with high attack rates; 30 cases of invasive pneumococcal disease/100,000 carriage acquisitions were detected (*2*). Conversely, serotype 15A pneumococci show low invasive potential and are usually associated with ST63 and resistance to macrolides, clindamycin (*ermB*) and tetracycline (*tetM*) (*2**,**16*). The overall frequency of clonal complex (CC) 63 among invasive isolates from adults was low: 2.5% (22/899) in 1997–2008 (*4*). Moreover, in 3 studies conducted in Spain with quinolone-resistant and macrolide-resistant pneumococci, CC63 was 1 of the most prevalent clones (*16**,**19**,**22*).

The results of the present study suggest that the new strain could be the result of a recombination event between a serotype 15A-ST63 pneumococcal isolate that contained the S79F change in ParC and a serotype 8-ST53 pneumococcus. In this event, the recombination fragment included the *pbp2x* gene and part of the *pbp1a* gene. The recombination of the *cps* locus and flanking regions has been observed by other authors, (*6**,**24**,**25*), which suggests that it is a common biologic process in the evolution of pneumococci.

The new strain, serotype 8-ST63, is penicillin susceptible (MIC <0.01 μg/mL), and ST63 isolates with other serotypes (15F, 15A, 15B, 19F, and 19A) are usually penicillin resistant (MIC range 0.12 μg/mL−0.5 μg/mL) (*10**,**16**,**22*). The penicillin resistance of CC63 isolates is associated with a Q552E change in PBP2X (*26*). The acquisition of *pbp2x* genes from serotype 8-ST53, without changes involved in β-lactam resistance, by the recombinant strain explains its penicillin susceptibility.

There have been other examples of recombinant clones caused by capsular switching that successfully spread. For instance, in the past decade, acquisition of serotype 14 by pneumococci of serotype 9V of ST156 has been associated with the worldwide increase of serotype 14 as a cause of invasive pneumococcal disease (*4**,**6*). Moreover, exchange of capsular genes with *pbp* genes has been documented in the serotype 19A-ST320 clone that spread worldwide and is a major cause of multidrug-resistant invasive and noninvasive disease (*27**,**28*). In the United States, recombination of capsular and *pbp* genes has been observed as a vaccine escape of a penicillin-susceptible serotype 4 that acquired the capsular genes and PBPs of a serotype 19A strain. This recombinant strain had a nonvaccine serotype and an antimicrobial drug resistance pattern that favored its spread in the United States. (*5*). To the best of our knowledge, the new recombinant clone (serotype 8-ST63) described here has not been detected outside Spain.

A report from South Africa described the spread of 2 quinolone-resistant strains that caused invasive disease and colonized children; most of these children were receiving treatment with antimicrobial drugs (including levofloxacin) for multidrug-resistant tuberculosis (*29*). Moreover, in previous studies, our group and others have observed some clustered infections, with a limited number of cases caused by quinolone-resistant pneumococci in a specific geographic area (*19**,**30*). The recombinant strain (serotype 8-ST63) described in the present study has been detected in several cities in Spain, which demonstrates its ability to disseminate.

Most isolates of this new clone with ciprofloxacin resistance required MICs of levofloxacin that were in the susceptible range according to recommendations of the Clinical and Laboratory Standards Institute (*12*). Because these strains had a first-step mutation, use of fluoroquinolones to treat infections could become a problem. At least 1 death, resulting from therapeutic failure of levofloxacin, has been documented: the patient had pneumococcal pneumonia caused by a serotype 8-ST63 pneumococci in Seville, Spain. In this case, the initial strain had the S79F change in ParC and required a levofloxacin MIC of 1 μg/mL. After levofloxacin therapy, high-level drug resistance developed in this strain after a second change in GyrA (*31*).

In the 1990s, use of antimicrobial drugs was high in Spain. During this period, penicillin-resistant and multidrug-resistant clones spread over the country, caused invasive disease, and colonized healthy children. Since that time, use of antimicrobial drugs has decreased in Spain (*32*). Together with vaccination with the 7-valent pneumococcal conjugate vaccine, the decrease in use of β-lactams probably contributed to the recent decrease in penicillin-resistance and multidrug-resistance rates (*4**,**8*). In contrast, data from the Spanish Medicines Agency (http//agemed.es) indicate that levofloxacin consumption has increased from 0.2 defined daily doses/1,000 persons/day (DDD) in 2002 to 0.4 DDD in 2006 and 0.6 DDD in 2012. This antimicrobial drug pressure could have favored spread of the fluoroquinolone-resistant clone in Spain and resulted in the increase in the proportion of HLCipR among serotype 8-ST63 isolates.

Children are the main reservoir for pneumococci, but quinolones are seldom prescribed for this population. Thus, isolation of quinolone-resistant pneumococci from children is rare. We have not detected any serotype 8-ST63 pneumococci in isolates from children. However, in a plausible scenario, the recombinant clone could colonize the nasopharynx of children and become a major source for dissemination.

In conclusion, emergence and spread of the serotype 8-ST63 clone that originated by genetic interchange of capsular genes and their flanking regions, including *pbp1a* and *pbp2x* genes, have been detected in Spain. The 2 clones involved in capsular switching were Netherlands 8-ST53 and Sweden 15A-ST63. Surveillance is needed to clarify the dynamics of this new multidrug-resistant clone as cause of pneumococcal disease.
